# DES-Mutation: System for Exploring Links of Mutations and Diseases

**DOI:** 10.1038/s41598-018-31439-w

**Published:** 2018-09-06

**Authors:** Vasiliki Kordopati, Adil Salhi, Rozaimi Razali, Aleksandar Radovanovic, Faroug Tifratene, Mahmut Uludag, Yu Li, Ameerah Bokhari, Ahdab AlSaieedi, Arwa Bin Raies, Christophe Van Neste, Magbubah Essack, Vladimir B. Bajic

**Affiliations:** 1 King Abdullah University of Science and Technology (KAUST), Computational Bioscience Research Center (CBRC), Thuwal, 23955-6900 Saudi Arabia; 2 King Abdulaziz University (KAU), Faculty of Applied Medical Sciences (FAMS), Department of Medical Laboratory Technology (MLT), Jeddah, 21589-80324 Saudi Arabia; 3 Ghent University, Center for Medical Genetics Ghent (CMGG), B-9000 Ghent, Belgium

## Abstract

During cellular division DNA replicates and this process is the basis for passing genetic information to the next generation. However, the DNA copy process sometimes produces a copy that is not perfect, that is, one with mutations. The collection of all such mutations in the DNA copy of an organism makes it unique and determines the organism’s phenotype. However, mutations are often the cause of diseases. Thus, it is useful to have the capability to explore links between mutations and disease. We approached this problem by analyzing a vast amount of published information linking mutations to disease states. Based on such information, we developed the DES-Mutation knowledgebase which allows for exploration of not only mutation-disease links, but also links between mutations and concepts from 27 topic-specific dictionaries such as human genes/proteins, toxins, pathogens, etc. This allows for a more detailed insight into mutation-disease links and context. On a sample of 600 mutation-disease associations predicted and curated, our system achieves precision of 72.83%. To demonstrate the utility of DES-Mutation, we provide case studies related to known or potentially novel information involving disease mutations. To our knowledge, this is the first mutation-disease knowledgebase dedicated to the exploration of this topic through text-mining and data-mining of different mutation types and their associations with terms from multiple thematic dictionaries.

## Introduction

Links between mutations and diseases are not restricted to rare diseases, as associations between common diseases (such as cancer, heart disease, diabetes etc.) and genetic variants (mutations) were found and shown to influence susceptibility to these diseases too^1^. Thus, tools such as PVP^2^, GWAVA^3^, CADD^4^, DANN^5^, FATHMM-MKL^6^ were developed to identify pathogenic and causal mutations in the human genome. However, association studies coupled with the development of these tools, added even more evidence to the plethora of mutation-disease information in the published literature. Specifically, in the past two decades alone, more than 2,500 publications related to genome-wide association studies (GWAS) were published in over 300 different journals^7^. The large volume of data generated from these studies further prompt the development of resources focused on mutation-disease. However, the well-established resources such as OMIM^8^, dbSNP^9^, HGMD^10^, ClinVar^11^, BioMuta^12^, MutDB^13^, SNPedia^14^, UniProt^15^ and Variome^16^ that incorporate such information require sifting through large amounts of data to localize the information of interest. Each of these resources has a different number of mutation-disease information and harbor different levels of detail. For example, in the OMIM database, 5,074 genetic diseases are associated with one or more mutations, while in SNPedia only 463 diseases are associated with mutations. Nonetheless, these public databases only contain a subset of mutation-disease associations that exist in the literature, because extracting all is tied to problems due to nomenclature complexity and the need for a significant level of manual curation.

To address some of these issues, several text-mining-based mutation detection tools (such as MutationFinder^17^, SETH^18^, and tmVar^19^) and tools that find links between mutations and genes and/or diseases (such as Dimex^20^, EMU^21^, PubTator^22^, and PolySearch^23^) have been developed. These tools are based on algorithms that sift through biomedical text to detect mutations. It is common to use standard regular expressions to identify either only point mutations, such as in the case of MutationFinder, or multiple mutation types using conditional random fields as in tmVar or named entity recognition of genetic variants using Extended Backus-Naur Form grammar as in SETH. Other tools, such as EMU and Dimex, also detect the gene and diseases associated with mutations. EMU extracts this mutation-gene-disease association using a rule-based method, while Dimex extracts the same associations using a Natural Language Processing-based mining method. Apart from the mutation detectors, other related tools, such as PubTator and PolySearch, also provide mutation-related information based on text mining. PubTator is a web-based system that assists in biocuration by deploying several entity recognition tools including tmVar for mutations, DNorm^24^ for diseases, GeneTUKit^25^ for gene mentions and GenNorm^26^ for gene normalization. On the other hand, PolySearch uses a co-occurrence-based text-mining approach to extract relationships between human diseases, genes, mutations, drugs and metabolites. When the connection between the mutations and genes are found, one can benefit from using the DAVID^27^ system to determine likely links of mutations to diseases based on gene enrichment for different diseases. As yet, none of these resources has combined, 1/text-mining the entire PubMed and available PMC full text articles, with 2/providing comprehensive associations of mutations to terms from 26 other topic-related terms including diseases, genes, metabolites and drugs, where terms are found to be statistically enriched in mutation-disease related literature, and 3/providing such information for multiple mutation types.

To overcome some of these limitations, we developed the mutation-focused knowledgebase (KB), DES-Mutation, based on the methods and concepts applied to similar topic-specific KBs^28–42^. DES-Mutation makes use of precompiled dictionaries that contain the terms used to index the text from both PubMed (title and abstract) and PubMed Central (PMC) (full text) articles. In this manner, DES-Mutation links human mutations with different categories of terms such as human diseases, human genes, pathogens, toxins, etc., that are enriched in mutation-disease literature. The system allows for exploring the context of mutation-disease links that no other system provides. DES-Mutation provides a platform that allows users to explore statistically enriched co-occurring terms and potential hypotheses. We provide illustration of how DES-Mutation can be used to assist research in the mutation-disease domain. To our knowledge, this is the first mutation-disease knowledgebase dedicated to the exploration of this topic through text-mining and data-mining of different mutations types and their associations with terms from multiple thematic dictionaries.

## Results and Discussion

**Figure 1:**
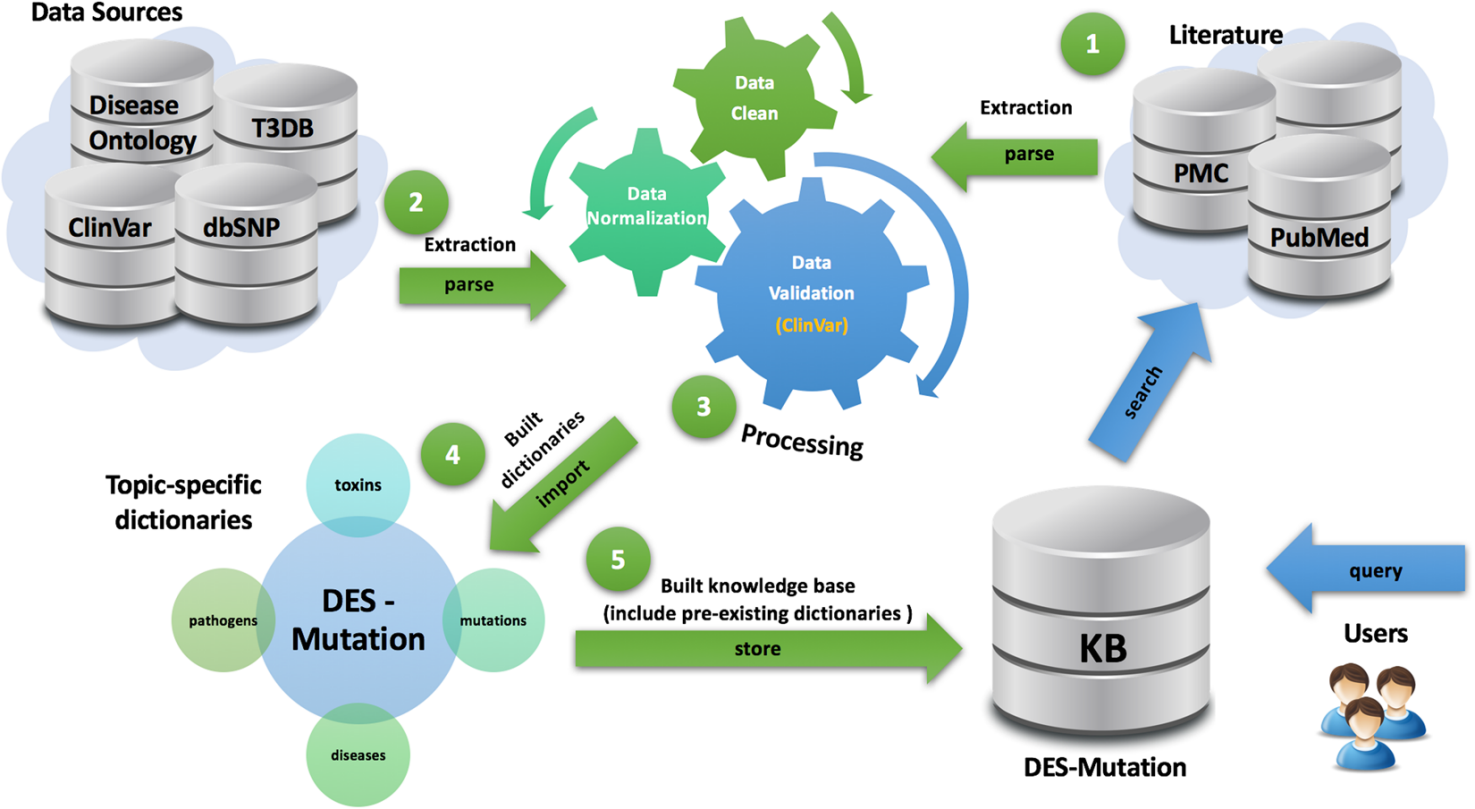
Workflow used to construct the DES-Mutation KB.

The process used to construct the DES-Mutation KB is depicted in Fig. 1. In the Methodology section, we provide a detailed description of the information-mining approach used. Briefly, we queried terms related to mutations and retrieved 436,257 PubMed and PubMed Central (PMC) articles from which we extracted term-document mapping information using 27 different dictionaries relevant to this topic-specific KB. We then analyzed this information for statistically enrichment of terms, enrichment of pairs of terms, and integrated these data with relevant external resources to create the DES-Mutation KB.

### Development of dictionaries incorporated into DES-Mutation

Of the 27 different dictionaries used in this KB, seven dictionaries (“ATC Ontology (Bioportal)^43^”, “EGO Ontology (Bioportal)^44^”, “HP Ontology (Bioportal)^45^”, “ICD9^46^”, “ORDO Ontology (Bioportal)^47^”, “PDO Ontology (Bioportal)^44^”, and “Mutations (tmVar)”) were newly compiled (Tables 1 and 2) to ensure relevance and comprehensiveness of the theme-specific topic. In Table 1, the other 20 dictionaries used in this work are denoted as “pre-existing in DES” as they were previously created for use in other knowledgebases developed using the DES system/framework. The “Drugs (DrugBank)” dictionary is updated. The compilation of most of the new dictionaries followed the standard process^36^, except for the “Mutations (tmVar)” dictionary. Reason being, there are currently over 170 million human SNP records in dbSNP (ver. 150), and inclusion of all the SNPs into the KB would make KB extremely slow and thus of no interest to end users. However, since majority of these SNPs have not been mentioned in publications, we used a machine learning based text-mining mutation extraction tool on the entire PubMed and PMC. This enabled us to find all the SNPs from the dbSNP database as well as SNPs outside of the dbSNP, as long as these have been mentioned in the publications. A comparison of the existing mutation detector tools, has shown^48^ that tmVar achieves the same or higher performance in precision, recall and F-measure than MutationFinder, EMU and SETH (using the MutationFinder dataset). It further shows that tmVar also achieves the higher performance in terms of recall and F-measure than SETH (using the tmVar dataset), but SETH achieved the higher performance in terms of precision. Thus, we used tmVar to extract the sequence variants (using the Human Genome Variation Society (HGVS) nomenclature) from all PubMed (abstracts) and PMC (full-text) documents (see the Methods section). Note that we only used the portion of PMC documents allowed by copyright for text-mining. The tmVar platform extracted 628,013 potential mutation mentions from the literature. Table 3 shows different mutation types extracted.

**Table 1:** List of dictionaries used in DES-Mutation. References for the data sources indicated are as follows: Bioportal, ChEBI^71^, Entrez Gene^72^, MetaboLights^73^, IntEnz^74^, T3DB^75^, Industrially Important Enzymes^76^, GO^77^, KEGG^78^, Reactome^79^, PANTHER^80^, UniPathways^81^, NCBI Taxonomy^82^, KOBAS^83^.

Dictionary	Enriched Unique Terms in the KB	Source
**Chemicals/Compounds**		
Antibiotics	633	pre-existing in DES
Chemical Entities of Biological Interest (ChEBI)	19,988	pre-existing in DES
Drugs (DrugBank)	4,100	updated
Lipids	2,406	pre-existing in DES
Toxins (T3DB)	1,908	pre-existing in DES
Functional Annotation		
Biological Process (GO)	7,716	pre-existing in DES
Cellular Component (GO)	1,913	pre-existing in DES
Molecular Function (GO)	2,640	pre-existing in DES
Pathways (KEGG, Reactome, UniPathway, PANTHER)	1,925	pre-existing in DES
**General**		
ATC Ontology (Bioportal)	1,838	newly compiled
DOID Ontology (Bioportal)	5,147	pre-existing in DES
EGO Ontology (Bioportal)	1,047	newly compiled
HP Ontology (Bioportal)	5,285	newly compiled
Human Anatomy	3,345	pre-existing in DES
ICD9	821	newly compiled
ORDO Ontology (Bioportal)	7,361	newly compiled
PDO Ontology (Bioportal)	286	newly compiled
**Genes/Proteins/Transcripts**		
Archaea Genes (EntrezGene)	6,687	pre-existing in DES
Bacteria Genes (EntrezGene)	45,120	pre-existing in DES
Fungi Genes (EntrezGene)	19,173	pre-existing in DES
Human Genes and Proteins (EntrezGene)	25,617	pre-existing in DES
Mutations (tmVar)	63,413	newly compiled
Viruses Genes (EntrezGene)	6,203	pre-existing in DES
**Taxonomy**		
Archaea (NCBI Taxonomy)	761	pre-existing in DES
Bacteria (NCBI Taxonomy)	13,496	pre-existing in DES
Fungi (NCBI Taxonomy)	7,162	pre-existing in DES
Viruses (NCBI Taxonomy)	4,883	pre-existing in DES

**Table 2:** Ontologies used in creation of new dictionaries incorporated in DES-Mutation.

Ontology	Description
ATC Ontology	**Anatomical Therapeutic Chemical Classification Otology:** a representation of the ATC classification provided by WHO used for the classification of drugs.
DOID Ontology	**Human Disease Ontology**^84^**:** a comprehensive hierarchical representation for human disease.
EGO Ontology	**Epigenome Ontology:** a biomedical ontology for integrative epigenome knowledge representation and data analysis.
HP Ontology	**Human Phenotype Ontology:** a representation of human phenome annotations with monogenic diseases listed in the Online Mendelian Inheritance in Man (OMIM) database.
ORDO Ontology	**Orphanet Rare Disease ontology:** a representation of rare diseases capturing relationships between diseases, genes and other relevant features which will form a useful resource for the computational analysis of rare diseases.
PDO Ontology	**Pathogenic Disease Ontology:** a representation of human infectious diseases caused by microbes and the diseases that is related to microbial infection.

**Table 3:** tmVar: Number of detected entries belonging to different kind of mutations based on the PubMed and PMC.

Mutation Category	Number
SNP	19,899
Substitution	588,210
Deletion	12,483
Insertion	3,583
Duplication	773
InDels	295
Frameshift	2,770

Although tmVar extraction algorithm uses the HGVS convention, it still gives a number of false positives. For example, it extracts strings that do not have a location or has a negative location. To solve this issue, we develop an in-house script containing a list of rules and then we apply it on all the potential mutations extracted by tmVar. For that cleaning, we used frequency threshold, pattern matching, and manual verification on the data. For the frequency threshold we applied the following rule: all mutations with frequency more than 50,000 are removed. This was decided after observing that most hits above this threshold were false positives containing mostly short patterns like ‘A > C’. We would like to mention again, that tmVar applies a normalization to the results using the HGVS convention, but some of the results were missing some of the fields in the normalized form (such as the location of the mutation). For this reason, our pattern matching script followed some specific rules: 1) delete unreasonable mutation extractions (such as alphabetic characters in the position field) 2) check if all detected mutations (using HGVS convention) contain all the fields. Hence, for a mutation detected as Substitution, it needs to include the following information: the sequence type, the mutation type (SUB), the wild type, the mutation position, and the mutant. For example, hits normalized to |SUB|A|| would be deleted. After this cleaning, out of all the mutations that tmVar extracted, only 105,511 unique normalized mentions remained (19,899 having an rs/ss identifier and 85,612 being other types of mutations). Using this cleaning process, we decreased the number of false positives generated by tmVar. Next, to see if these mutations are valid, we performed validation by two methods, first by validating the list of potential mutations with ClinVar for the 85,612 mutations, and second, via dbSNP for the 19,899 mutations with an rs identifier. Since ClinVar is biased towards mutations which have been assigned an rs identifier, we needed another way to verify that the extracted term is true mutation. With ClinVar and dbSNP we validated 54,899 and 16,475 mutations respectively (71,374 in total). Out of these, 63,488 mutations were enriched in the text analyzed and incorporated into the “Mutations (tmVar)” dictionary of DES-Mutation.

### Basic features of resources that contain mutation-disease associations extracted via text- mining

To the best of our knowledge, DES-Mutation is the first KB that contains multiple classes of mutations and their association with diseases and 25 other categories of relevant terms as extracted via text-mining from a large number of PubMed and PMC documents. The resource we found to be most similar to DES-Mutation are PubTator and PolySearch. PubTator extracts mutation-disease associations from PubMed abstracts only for multiple types of mutations. PolySearch extracts mutation-disease associations from both PubMed abstracts and PMC full text articles, but mutations are only point mutations. Contrary to these, DES-Mutation extract mutation-disease association from both PubMed abstracts and PMC full text articles and for multiple types of mutations. Also, PubTator and PolySearch highlight other terms in the text beyond mutations related to disease, genes, chemicals, and species, irrespective if these terms are enriched in the analyzed text or not, whereas DES-Mutation provides a means to explore much more topic-related statistically enriched terms beyond diseases, genes, chemicals and species, such as pathways, pathogens, etc. (see “Knowledgebase Statistics” section). In Table 4, we compare DES-Mutation to other similar text-mining based resources. Not all resources provide clear statistical information regarding their content, thus information provided is based on their original publication or information available on the web site.

**Table 4:** Comparison of select resources that provide mutation-disease associations extracted via text-mining.

	Topics	Online text-mining recourses		
		PolySearch	PubTator	Des-Mutation
Literature	Full-text (PMC)	+	−	+
	Abstract (PubMed)	+	+	+
Biomedical entities	Mutations	SNPs	SNPs, Sub, Del, Ins, Dup, FS, InDels	SNPs, Sub, Del, Ins, Dup, FS, InDels
	Genes	+	+	+
	Diseases	+	+	+
	Toxins	+	−	+
	Drugs	+	+	+
	Chemicals	+	+	+
	Species	+	+	+
	Proteins	+	+	+
	Metabolites	+	−	+
	Pathways	+	−	+
	Organs/tissues	+	−	+
	Taxonomies	+	−	+

### Knowledgebase Statistics

A total of 249,812 concepts are found enriched in the KB. We have in total 63,413 mutations found in the literature corpus being significantly enriched (FDR < 0.05) that resulted in 56,811 statistically enriched associated pairs between Mutations and Diseases (DOID Ontology (Bioportal)). Table 1 illustrates the DES-Mutation dictionaries. Table 2 contains the description of the Ontologies that are being used from DES-Mutation.

### Assessment of the quality of information extracted by the DES-Mutation system

It is difficult to provide a global assessment of the quality of extracted information by the DES-Mutation system. However, here we provide an independent assessment by comparing the quality of a subset of information extracted by DES-Mutation with that of EMU (used for mutation mentions) and ClinVar (used for mutation-disease associations). EMU was chosen because at its core it is a rule-based mutation detection tool that employs regular expression patterns to identify mutation mentions in text. Then subsequently, EMU incorporates extra step to associate mutations to genes and diseases. In particular, it utilizes a detect-filter-assert approach, whereby a set of positive patterns are used to harness mutation hits, and these are then filtered using a set of fallible/negative patterns in order to reduce false positives, such as cell-line names. To associate the mutations to genes, DES-Mutation extracts gene names/symbols using a dictionary-based term matching approach; then the amino acid sequences of proteins encoded by these genes are fetched from GenBank^49^ and used to ascertain whether the mutations co-occurring in the same text can actually occur in the mentioned genes. The association between a gene and a mutation mentioned is validated when there is a match for the amino acid at the position of the mutation for at least one of the associated proteins. EMU’s method for associating these validated mutations to diseases is, however, not as rigorous, namely it is to merely use corpora specific to the particular disease in question. For example, to extract mutations related to prostate cancer (PCa), and breast cancer (BCa), a corpus relevant to these two diseases was used^21^.

It is worth noting, however, that within a corpus relevant to a particular disease (such as PCa), a substantial number of other diseases can be discussed for various reasons, including comparison, association, or as background examples. The same can be said for gene and mutation mentions within these corpora. EMU does not provide an extra step to actually validate the mutation-disease association as is done with genes. The other issue to note, is that validating mutations through sequence analysis, might increase the precision of extracted mutations, however, it affects recall substantially even though these associations are not conclusive anyway, and have to eventually be curated. Frequently are mutations mentioned without the actual genes being present in the same abstract, and useful information can still be derived by these mutation hits. These are two key differences with DES-Mutation approach to concept association. DES-Mutation associates concepts through statistical enrichment as explained in the Methodology section, thus increasing the probability of reported associations to be meaningful. However, that meaning is left to the user to decide on by looking at the actual related literature (easily accessible from the interface). So, if a number of genes keep co-occurring with a particular mutation much more frequently than is statistically expected by mere random chance, then these genes are deemed important to the context of discussing that mutation or vice-versa. The users can see this, and follow up on the link if they so wish. This is also due to the fact that DES-Mutation is a system that exposes a substantial amount of information from a voluminous corpus, annotated with many biomedical entity dictionaries. Because of the above fundamental disparity in the premise of each system, the following comparison between DES-Mutation and EMU will focus mainly on the quality of extracted mutations (excluding the sequence filter step in EMU). Thus, we here provide a comparison of DES-Mutation and EMU annotations for mutations mentions in randomly selected abstracts.

#### Comparing DES-Mutation with EMU (mutation mentions)

For this comparison, 1000 abstract-only documents were randomly selected from the DES-Mutation annotation to be the basis of comparison. These documents were also used as input for the EMU mutation extractor (the Perl scripts for the EMU extractor can be found at http://bioinf.umbc.edu/EMU/ftp/). The two resulting mutation annotations differ in their format (see Tables 5 and 6), so necessary pre-processing was performed to allow for the comparison:

**Table 5:** DES-Mutation annotation example (follows the tmVar style of normalization).

**PMID**	**Mutation Mention**	**Count**	**Normalized Form**	**Type**
2746736	lysine to methionine at position 227	1	p|SUB|K|227|M	Protein Mutation
12782315	Y151M	3	p|SUB|Y|151|M	Protein Mutation
23084080	rs4986790	4	rs4986790	SNP: rs4986790

**Table 6:** EMU annotation example.

**PMID**	**Mutation**			**Level**	**Type**	**Mention**	**Position**
7528778	SER	GLY	325	PROTEIN	MISSENSE	serine to glycine	position 325
15896662	PRO	ARG	249	PROTEIN	MISSENSE	P249R	
23989986	rs6094710	—	—	ALL	RSID	rs6094710	—

In particular, an equivalence relationship between the two annotations had to be established to allow for easy mapping across the normalized forms. This is due to the fact that trying to map at the level of the mention may prove futile, because the systems employ radically different ways of extraction, so the delimiters of the mutation mention are rarely aligned. Therefore, the annotations were decomposed into: RS# type identifiers, and non-RS# mutations. The latter were decomposed into DNA level, and protein level mutations. Both annotations contained DNA-indel as well as DNA-missense type mutations. For protein level mutations, all EMU hits were missense hits, while DES-Mutation also had protein-indel, as well as protein-FS (Frame Shift) type mutations (see Table 7).

**Table 7:** Comparison of DES-Mutation and EMU annotations for mutation mentions in 1000 randomly selected abstracts from the DES-Mutation corpus. There are 86% of DES-Mutation hits that are validated by EMU. The union annotation of DES-Mutation and EMU is 84% covered by DES-Mutation hits. ‘\’: is the set difference operator; ‘∩’: is the set intersection operator, ‘∪’: is the set union operator; DES_hits_\EMU_hits_: hits exclusive to DES-Mutation; EMU_hits_\DES_hits_: hits exclusive to EMU; DES_hits_ ∩ EMU_hits_: hits common to DES-Mutation and EMU; DES_hits_ ∪ EMU_hits_: union of both annotations.

	**DES**_**hits**_	**DES**_**hits**_\**EMU**_**hits**_	**DES**_**hits**_ ** ∩ EMU**_**hits**_	**EMU**_**hits**_\**DES**_**hits**_	**EMU**_**hits**_	**DES**_**hits**_ ** ∪ EMU**_**hits**_
DNA - indel	68	28	40	38	78	**106**
DNA - missense	288	70	218	212	430	**500**
Protein - indel	14	14	0	0	0	**14**
Protein - missense	1535	178	1357	116	1473	**1651**
Protein - FS	7	7	0	0	0	**7**
RS #	203	2	201	43	244	**246**
Total	**2115**	**299**	**1816**	**409**	**2225**	**2524**

As we do not have a “gold standard” for this comparison, we can either trust both systems in terms of having 0 false positives, in other words the union annotation is the set of positives. Or we can only trust the intersection to be the set of positives, disregarding exclusive hits as false. We also include a strict case whereby we only consider all of EMU hits to be positives, and anything else as a negative. Using these cases, we measure the performance of the DES annotation in terms of Recall, Precision and F-measure.

##### **Case 1:**

 reference is the union of hits of DES and EMU against the 1000 randomly selected abstract-only documents (A\B is the set difference of sets A and B):

FP = 0, FN = EMU_hits_**\**DES_hits_ = 409, TP = 2115,

**Precision** = **100%, Recall** = **84%, F-measure = 91%**.

##### **Case 2:**

 reference is the intersection of hits of DES and EMU against the 1000 randomly selected abstract-only documents:

FP = DES_hits_**\**EMU_hits_ = 299, FN = 0, TP = 1816,

**Precision = 86%, Recall = 100%, F-measure = 92%**.

##### **Case 3:**

 reference is the set of hits of EMU against the 1000 randomly selected abstract-only documents:

FP = 299, FN = 409, TP = 1816:

**Precision = 86%, Recall = 82%, F-measure = 84%**.

Note that in the above, DES-Mutation recall is slightly negatively affected due to the statistical enrichment cut-off applied to concepts. Nonetheless, these results show that EMU and DES-Mutation are comparable in terms of their coverage and quality of extracted mutation mentions. However, while EMU attempts to validate mutation-gene associations through sequence analysis, DES-Mutation focuses on providing an explorative interface to users, where they can investigate different types of associations of terms from various dictionaries, and validating them by referring to linked literature.

#### Comparing DES-Mutation with ClinVar (mutation-disease associations)

For assessing accuracy of mutation-disease associations suggested by DES-Mutations, we used ClinVar data. For this purpose, we did not use EMU because its accuracy results have been obtained based on preprocessed data where, for example, inconclusive entries and entries where curators did not agree have been removed. Our results, however, are based on the raw unprocessed data. Thus, we analyzed 600 mutation-disease associations suggested by DES-Mutations. These 600 associations are provided in Supplementary Table S1, with relevant associated information. Our system extracted 437 correct associations out of all 600 associations, resulting in *Precision* = 72.83% (437/600). We also observed that when the extracted association is found in several articles, the precision is higher. For example, in cases when there are 8 or more supporting articles, the precision is over 80%. For example, EMU achieved precision of 55% and 77% on significantly smaller breast cancer and prostate cancer data, respectively. The 600 associations represent all predicted correct cases (i.e., the sum of all correct and false correct associations). Out of the correctly extracted associations, there are only 41 associations (9.38% = 41/437) common with the ClinVar entries. The remaining 396 are novel, not present in ClinVar. Thus, out of correctly extracted associations, 90.62% (396/437) are novel. These novel associations make 39.6% of all extracted associations. The small overlap with the ClinVar data is understandable as both ClinVar and DES-Mutation contain only partial information about mutation-disease associations.

Our system extracted 163 false positive associations. Many of the false associations were association with different disease mentioned in the text, or the symbol that should represent a disease actually represent a gene or a protein (as an example, NS1 refers to nonstructural protein 1 (NS1) in Influenza virus, instead of Noonan syndrome 1 (also known as NS1) and mutation is wrongly associated with NS1).

## Knowledgebase Utilities and Case Studies

DES-Mutation allows users to easily explore mutation-related literature based on statistically enriched terms and associations of these terms. These enriched terms can be explored in several contexts via links (described in^36^). Briefly, users can explore mutation-related information via enriched terms using the “Enriched Terms” [Concepts] link and enriched co-occurring terms (in the title/abstract level and in full-text document at the sentence level) using the “Enriched Term Pairs” [Associated Concepts] link. Users can explore if enriched term pairs are known or novel via the new “Explore Hypotheses” link. Links to “GO Enrichment”, “Reactome Enrichment”, “KOBAS Pathways”, “KOBAS Diseases”, “Import Associations” and “Literature” are also provided. “Help” tabs with ‘how to use’ instructions are provided for each link. To further facilitate the easy exploration of literature, enriched terms can be restricted via ranking options such as false discovery rate (FDR) calculated based on the Benjamini-Hochberg algorithm, etc. Additionally, each term has a hover box through which “Network”, “Term Co-occurrences”, and “Term Link Sources” links for the term of interest that can be further explored. Users are also provided a detailed downloadable “Software Manual” and a short introductory video showing basic functionalities of DES-Mutation on the “Home” page.

The possibility to generate multilayered networks of associated biomedical entities is a unique feature of DES-Mutations. This allows for exploring the context in which mutation-disease association appears and opens different insights into how different biomedical entities may affect mutation-disease association. This property is exploited in the examples below.

### Case studies that demonstrate how DES-Mutation can be used as a research support system

**Example 1:** Using DES-Mutation to find potentially novel mutation-disease associations.

**Figure 2:**
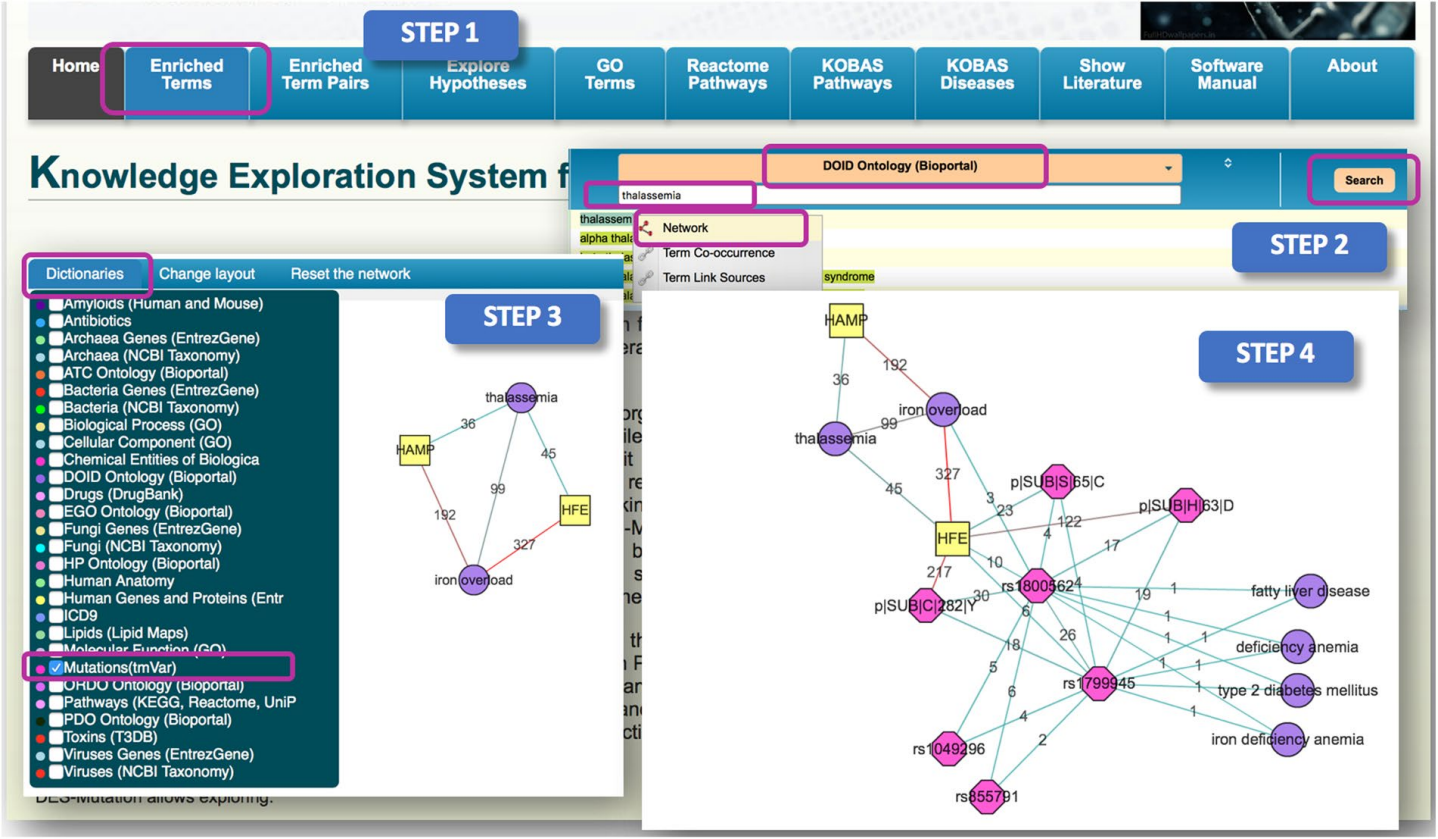
Step-by-step illustration of how DES-Mutation can be used to identify relationships between the human disease ‘thalassemia’ and mutations. The purple circles represent the “DOID Ontology (Bioportal)” dictionary; the yellow square represent the “Human Genes and Proteins (EntrezGene)” dictionary; and the pink octagons represent the “Mutations (tmVar)” dictionary. The edge color is distributed across a color spectrum from red (strong association) to blue (weaker association) based on the frequency of co-occurrence. The number on each edge represents the number of publications that link the associated nodes.

To explore such potential associations, we start by selecting the “Enriched terms” link (Fig. 2, Step 1). This opens a page that displays all enriched terms in the KB. However, since we are interested in the inherited blood disorder Thalassemia, we select the “DOID Ontology (Bioportal)” which is the Disease Ontology and we filter the dictionary with ‘**thalassemia**’. From ‘**thalassemia**’ we generate a ‘**Network**’ using the right click menu (Fig. 2, Step 2). To expand its associations, we clicked on ‘**Dictionaries**’ and checked on the “DOID Ontology (Bioportal)” and “Human Genes and Proteins (EntrezGene)” dictionaries. Then, we highlighted ‘**thalassemia**’ node and used its right click menu to ‘Expand from the term’. Here we found several known blood related disorders (such as **‘hemolytic anemia’, ‘anemia’, ‘hemoglobinopathy’, ‘sickle cell trait’**), iron-related disorder (**‘iron overload’**) and more general disease categories (such as**’cystic fibrosis’, ‘genetic disorders’, ‘genetic disease’, ‘muscular dystrophy’ and ‘Duchenne muscular dystrophy’**). Because **‘iron overload’** is a known characteristic of **‘thalassemia’**, we chose to similarly expand from the **‘iron overload’** node by checking on the “Human Genes and Proteins (EntrezGene)” dictionary only. The resulting network was simplified by removing all nodes with a single link (Fig. 2, Step 3). Here, we found that **‘HFE’** and **‘HAMP’** genes were linked to both the ‘**thalassemia**’ and ‘**iron overload**’ nodes. We then only expanded from the **‘HFE’** node by checking on the “Mutations (tmVar)” dictionary only, then selecting ‘Expand from the term’. This produced two SNPdb ID (‘**rs1800562**’ and **‘rs1799945’)** that were both further expanded from by checking on the “Mutations (tmVar)” and “DOID Ontology (Bioportal)” dictionaries. Here, once again, the resulting network was simplified by removing all nodes with a single link and all dna level mutations in this case because they are also represented by the protein level mutations (Fig. 2, Step 4).

The most severe forms of Thalassemia (Thalassemia Major or Cooley’s Anemia) causes a life-threatening anemia that requires regular blood transfusions that lead to iron-overload^50–52^. Thus, the Network accurately illustrates the link between iron overload and several closely related diseases. Also, **‘HFE’** and **‘HAMP’** genes have been linked to the pathophysiology of **‘thalassemia’** via 45 and 36 PubMed articles, respectively (see ‘Network’) which shows how extensively these links have been researched. **‘HFE’** is known to regulate the production of proteins located on the surface of primarily liver and intestinal cells. One of the proteins regulated by **‘HFE’** is Hepcidin, the “master” iron regulatory hormone produced by the liver. Hepcidin (**‘HAMP’** gene) functions by modulating levels of iron absorbed from the diet and released from storage sites^53,54^. β-Thalassemia is characterized by low levels of hepcidin (**‘HAMP’**), the hormone that regulates iron absorption^55^.

Moreover, it has been reported that the most prevalent mutation in **‘HFE’**, C282Y **‘rs1800562’**, alters the **‘HFE’** protein structure preventing the formation of a disulfide bond in the α3 domain, abrogates β2-microglobulin association and cell surface expression of the protein, that may be sufficient to cause iron storage overload^56^. Also, the second mutation in the **‘HFE’** gene, H63D **‘rs1799945’**, was shown to increase iron overload in β-Thalassemia carriers^57^. These two allelic variants of **‘HFE’** (C282Y and H63D) were also shown to be significantly correlated with Hereditary hemochromatosis (HHC), a disorder of iron metabolism, characterized by increased iron absorption and deposition in the joints, pituitary gland, pancreas, heart and liver^58^. The third mutation in the **‘HFE’** gene, S65C **‘p|SUB|S|65|C’**, has only been implicated in a mild form of HHC^59^ but has not been implicated in β-Thalassemia. The mutation **‘rs855791’** is related with the TMPRSS6 gene and the mutation P589S **‘rs1049296’** is related to the transferrin (TF) gene. Genome-wide association studies have shown that the SNP **‘rs855791’**, which causes the MT2 V736A amino acid substitution, is associated with variations of serum iron, transferrin saturation, hemoglobin, and erythrocyte traits^60^. Both of these two mutations are connected to iron-related diseases^55,61^. In fact, the epistatic interaction between P589S **‘rs1049296’** in the transferrin gene (TF) and C282Y **‘rs1800562’** in the hemochromatosis gene (**‘HFE’**) result in the increased risk of cognitive impairment and Alzheimer’s and Parkinson’s diseases^62,63^.

Thus, DES-Mutation can generate a ‘bird’s-eye-view’ of potential connections between disease and mutation. As shown above, DES-Mutation suggests that even though there is no literature evidence for **“p|SUB|S|65|C”** (S65C) being connected to thalassemia, this mutation of **‘HFE’** gene is associated with **‘iron overload’** that is characteristic of **‘thalassemia’**. Thus, DES-Mutation links **“p|SUB|S|65|C”** (S65C) mutation to **‘thalassemia’** via both **‘HFE’** and **‘iron overload’**, making this a novel hypothesis of the link of the mutation to **‘thalassemia’**.

#### Exploring relationships between low cholesterol level and MTB infection

**Figure 3:**
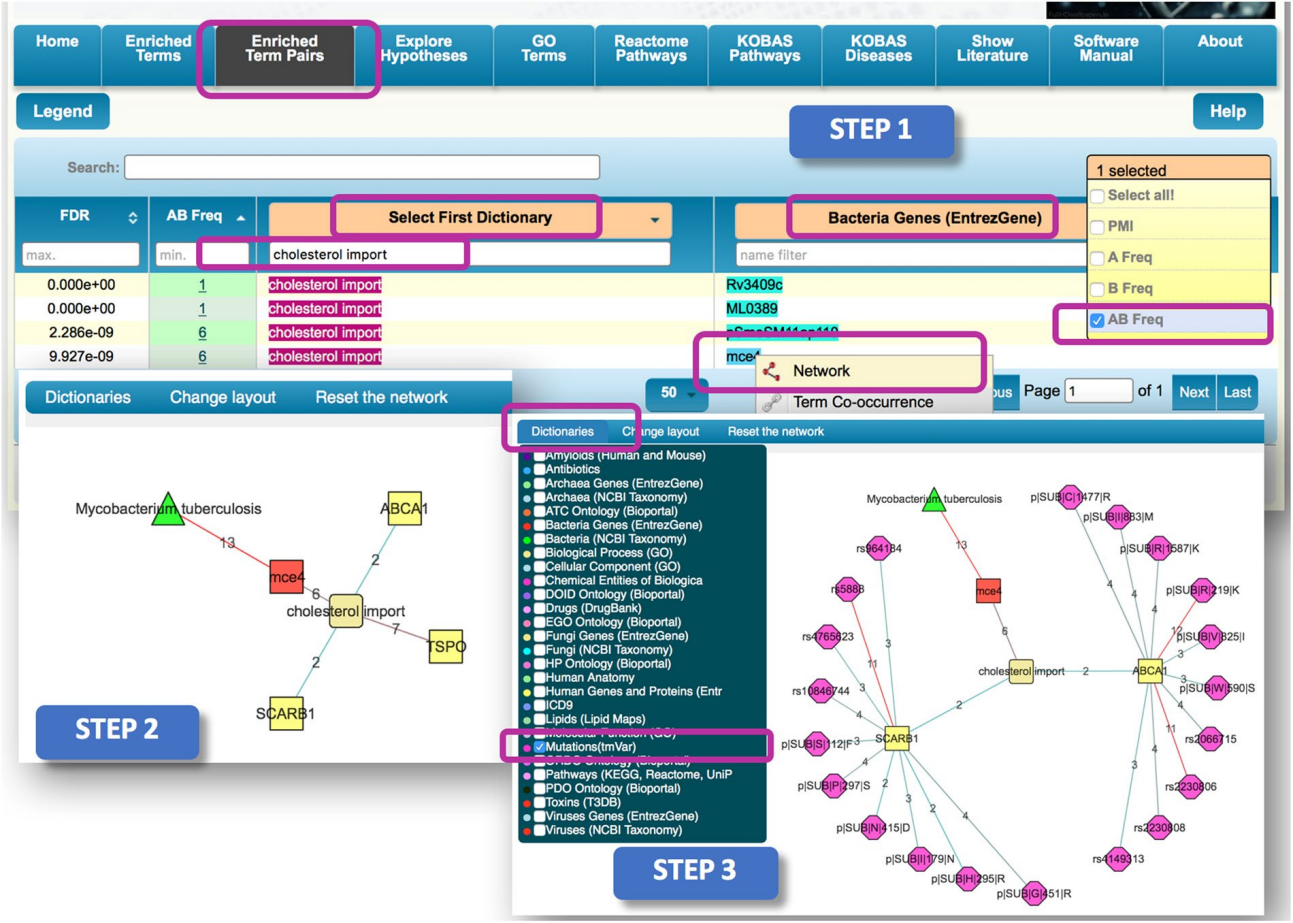
Step-by-step illustration of how DES-Mutation can be used to identify relationships between low cholesterol and MTB infection. The red square represents the “Bacteria Genes (EntrezGene)” dictionary; the green triangle represents the “Bacteria (NCBI Taxonomy)” dictionary; the yellow square represents the “Human Genes and Proteins (EntrezGene)” dictionary; and the pink octagons represent the “Mutations (tmVar)” dictionary. The edge color is distributed across a color spectrum from red (strong association) to blue (weaker association) based on the frequency of co-occurrence. The number on each edge represents the number of publications that link the associated nodes.

The higher the level of cholesterol the lower is the risk of the tuberculosis. In fact, hypocholesterolemia was shown to be a major risk factor for developing pulmonary tuberculosis^64^. However, exploring the underlying mechanism of this relationship is still in its infancy. Thus, to explore all possible relationship between cholesterol and MTB, using ‘Associated terms’ we filter the first column with biological process of interest, ‘cholesterol import’ and for the second column, we select the ‘Bacteria Entrez Genes’ dictionary. This process retrieves 4 records that represent terms that co-occur possibly suggesting a biological relationship. To increase the chances of unravelling a plausible hypothesis, we re-ordered the AB frequency column to display the ‘Bacteria Entrez Genes’ term with the highest number of publications linked to term A. This re-arrangement showed that **‘pSmeSM11ap110’** and **‘mce4’** have most links to term B. However, **‘pSmeSM11ap110’** is a locus tag identifier, so we did not consider it. From **‘mce4’** we generate a ‘Network’ using the right click menu (Fig. 3, Step 1). To expand its associations, we clicked on ‘Dictionaries’ and selected the “Bacteria (NCBI Taxonomy)” and “Biological Process” dictionaries, then highlighted **‘mce4’** node to use its right click menu to ‘Expand from the term’. Here, we found **‘Mycobacterium tuberculosis’** (MTB) and **‘cholesterol import’** nodes have the highest number of publication links to the **‘mce4’** node. Thus, the resulting network was simplified by removing all other nodes. The link between these nodes is expected, as previous studies demonstrated that the high level of mycobacterial persistence in its host, is partly due to MTB using the **‘mce4’** gene to acquire and utilize its hosts cholesterol as an energy and carbon source^65,66^. Moreover, the **‘mce4’** gene acts as a transporter in the MTBs’ cholesterol import system and loss of this function was shown to impair the growth of MTB predominantly during the chronic phase of murine infection^65^. Next, we expand the **‘cholesterol import’** node with ‘Human Genes and Proteins (Entrez Gene)’ dictionary (Fig. 3, Step 2). The resulting network was simplified in this case by removing all nodes with a single publication. This left us with the three genes including ‘**SCARB1**’, ‘**ABCA1**’ and **‘TPSO’**. However, since the normal function of **‘TPSO’** is associated with the reduction of cholesterol, we only expanded the remaining two genes (‘**SCARB1**’ and ‘**ABCA1**’) with terms from the ‘Human Mutations (tmVar)’ dictionary (Fig. 3, Step 3).

The network presents an overview of all plausible mutations that could be induced by **‘Mycobacterium tuberculosis’** (MTB) infection resulting in reduction of cholesterol. The network shows two human genes that are involved in maintaining the cholesterol level in the body, **‘ABCA1’** and **‘SCARB1’**, and a mycobacterial gene, **‘mce4’**. The **‘ABCA1’** acts as a key “gatekeeper” influencing intracellular **‘cholesterol transport’** while **‘SCARB1’** is a receptor for lipoproteins such as cholesterol. In a normal condition, the host **‘ABCA1’** gene will regulate the movement of cholesterol into the cell and the **‘SCARB1’** will bind to the cholesterol, acting as a receptor. Thus, we hypothesize that when there are mutations occurring in these genes, the host will display less resilience to MTB infection. Specifically, mutations in the **‘ABCA1’** and **‘SCARB1’** genes, may lead to the inability to control the “right balance” of cholesterol in the body and exacerbate cholesterol being imported into MTB. **‘SCARB1’** has been shown to facilitate the uptake of cholesteryl esters from high-density lipoproteins in the liver, which is supposed to reduce plague formation that could cause atherosclerosis. A number of recent studies have shown the links of atherosclerosis with MTB infection^67–69^. However, no known mutation associated with **‘SCARB1’** has been associated with lowering cholesterol. Nonetheless, the mutation **‘rs2230806’** in **‘ABCA1’** increases susceptibility to lowering of cholesterol levels.

As shown above, even though there is no literature evidence for mutation **‘rs2230806’** in **‘ABCA1’** gene being connected to MTB infection, DES-Mutation via connections to ‘cholesterol transport’ and the mycobacterial gene ‘mce4’, both being implicated in MTB infection, suggest this mutation’ potential association to MTB infection.

## Concluding Remarks

DES-Mutation provides various means that facilitate the easy exploration of mutation-disease relationships, based on terms and phrases enriched in published mutation-disease literature. These terms and phrases are provided in 27 topic-specific dictionaries (Table 1), which provide extensive insights into mutation-disease links. These insights are also not limited to point mutations but include seven different mutation types. Among the mutation-based resources, DES-Mutation has unique property of enabling network-based interpretation of context in which mutation-disease associations appear.

Nonetheless, DES-Mutation’s limitations are similar to most text-mining resources, as detailed in DES-TOMATO^70^, and some limitations are specific to this KB. For example, the toxin dictionary is currently limited to T3DB. Also, this KB cannot capture all associations due to inconsistencies in disease nomenclature, even though terms/phrases are normalized and several disease resources are used, for example, (ICD9, DOID Ontology, ORDO ontology, etc.). Also, limitations associated with Network generation include the following: 1) a term/node can only be expanded by a maximum of 10 sub-node, this was a design choice to avoid “hairball” networks and 2) an association between nodes does not specify the type of relationship giving the association, e.g., a mutation linked to multiple genes does not mean that mutation is found in all the genes but rather that the genes are discussed in the same context.

Nonetheless, the case studies demonstrate the usefulness of this KB despite these limitations. The user-friendly interface and extensive instruction manuals eases the information exploration process. The DES-Mutation KB will be updated biannually to include newly published articles and the “Mutations (tmVar)” dictionary will be expanded to include tmVar2.0.

## Materials and Methods

### Server architecture and underlying systems

DES-Mutation is a topic-specific literature exploration system, that is based on significantly improved text-mining and data-mining capabilities of the DES system for topic-specific literature exploration, that was originally developed by VBB and AR and used to create a number of KBs in past^28–37,42^. The knowledgebase is implemented and hosted on a CentOS-7 operating system. Results are provided using Apache web server version 2.4.6. A MongoDB (2.6.11) database stores the literature repository, and a PostgreSQL (9.2.15) database stores the KB index and related tables. Apache Lucene was used to index the documents. Various programming languages/tools were used to develop the KB including: Java (openjdk 1.8.0_91), PHP 5.4.16, JavaScript, JQuery 3.0.0 C/C ++ (gcc 4.8.5) and Perl v5.16.3. DES-Mutation is functional across commonly used web-browsers (Linux, Windows, and Mac OS platforms) and was specifically tested for Firefox, Chrome and Safari. The workflow used to construct the DES-Mutation KB is depicted in Fig. 1.

### Developing the dictionaries

To ensure relevant and comprehensive topic-specific information exploration, seven new dictionaries were developed/compiled to complement the 21 pre-existing DES v2.0 dictionaries we used for this study and listed in Table 1.

#### Mutation dictionary

The tmVar was used to extract the sequence variants using the Human Genome Variation Society (HGVS) nomenclature, from all PubMed (abstracts) and PMC (full-text) documents. The ClinVar and dbSNP datasets were used to validate the mutations extracted from the whole PubMed and PMC using the tmVar-based dictionary. We found that 83% of these SNPs exist in dbSNP dataset and 64% of all other mutation types exist in the ClinVar dataset.

#### Disease dictionary

In addition to the disease dictionary (DOID Ontology (Bioportal)), we compiled five novel disease-related dictionaries: 1) from WHO (ICD-9-CM), from NCBO Bioportal (ATC Ontology, HP Ontology, ORDO Ontology, and PDO Ontology). For all dictionaries, synonymous terms/phrases were normalized to ensure terms can be recognized through trusted sources such as EntrezGene ID, UniProt ID, NCBI Taxonomy ID.

### Preparing the literature corpus

PubMed and PMC articles, stored in our local literature repository (MongoDB), were queried using [(*mutation OR mutations OR indel OR indels OR deletion OR deletions OR insertion OR insertions OR mutagenesis*) *AND* (*human OR “homo sapiens” OR bacterium OR bacteria OR virus OR viruses OR fungi OR fungus*)] to create the DES-Mutation literature corpus on September 21, 2017. The query retrieved 458,158 articles that were used to build the KB, of which 266169 were full-articles and 191989 were abstract only documents. Out of these articles, 436,257 had annotations and are included in the KB.

### Selection of 600 gene-mutation associations extracted by DES-Mutation

To select gene-mutation associations as extracted by our system, we sorted pairs represented gene-mutation associations by the number of documents in which our system found them. Then we took all associations found on positions 101–200 (each supported by 16–27 publications), 401–500 (each supported by 8 publications), 901–1,000 (each supported by 5 publications), 2,901–3,000 (each supported by 3 publications), 4,901–5,000 (each supported by 2 publication) and 9,901–10,000 (each supported by 1 publication). In total, we used in this way 600 gene-mutation associations. These associations have been evaluated manually by curators, cross-checked and marked as correct by providing a PubMed ID of an article where the confirmation can be found. ‘N/A’ is used to denote false association, and it is accompanied by a comment why the association in false (see Supplementary Table S1).

#### Concept Enrichment

**Figure 4:**
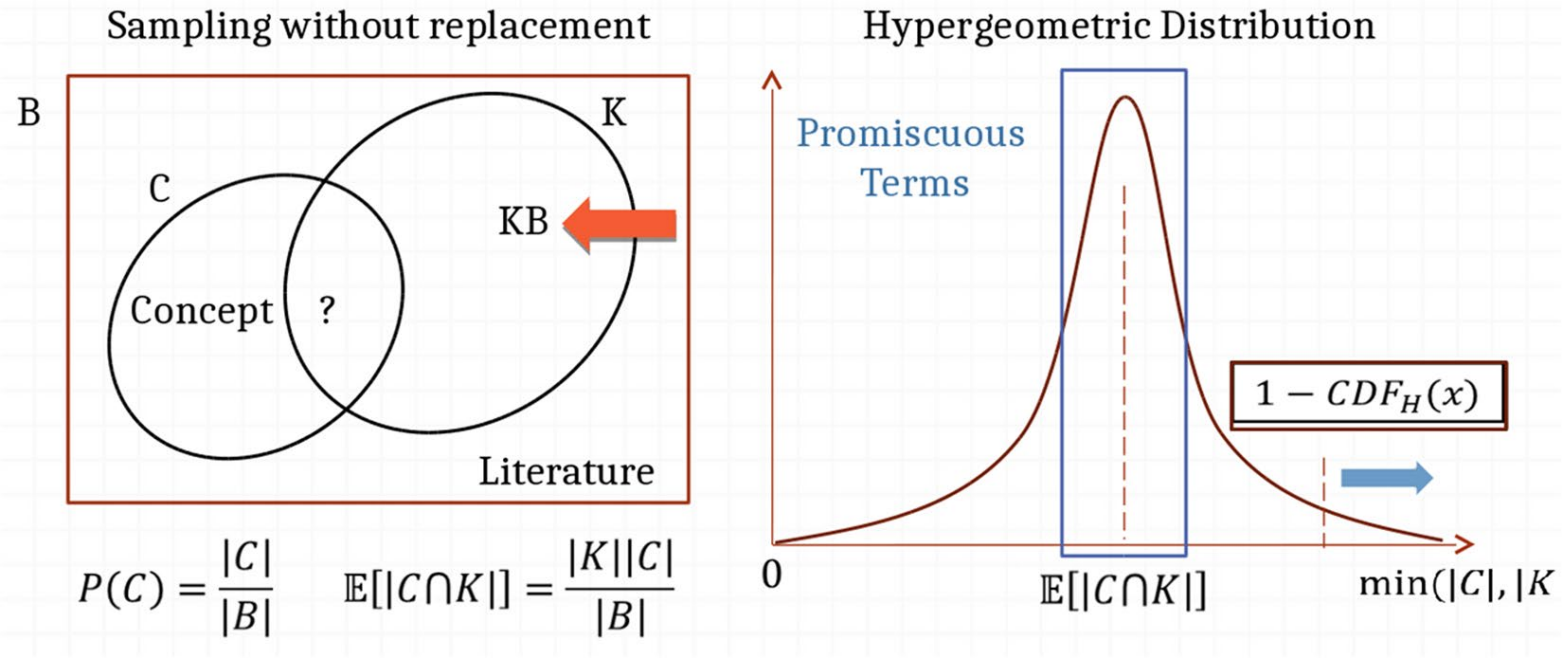
Concept Enrichment in DES.

If a concept occurs in the background annotation (the set of all articles in PubMed, and PMC, denoted as B) |C| number of times, then taking a random sample of documents, denoted as K, the concept has a probability P(C) = |C|/|B| to appear in each document, and its expected frequency in this random sample ${\mathbb{E}}[|{\rm{C}}\cap {\rm{K}}|]$ is proportional to the size of the sample K (namely ${\mathbb{E}}[|{\rm{C}}\cap {\rm{K}}|]\,=$ K × P(C)) (see Fig. 4).

If that sample in our knowledgebase results from a specific query however, we expect the query bias to affect observed concept frequencies, so they are not necessarily aligned with background ones. In other words, concepts relevant to the query would appear significantly more than their expectation of occurrence by random chance, while most irrelevant concepts would lie close to the mean of this hypergeometric distribution (if we consider sampling without replacement) or binomial distribution (if we consider sampling with replacement). Both types of modeling produce similar results for large enough KBs, but the hypergeometric distribution is more accurate for small KBs. In DES, concepts are enriched by computing the probability of observing them at least x number of times, where x is the actual frequency observation. This translates to computing 1-CDF(x) which is the complement of the cumulative distribution function at the observation x. This value gets smaller the more the concept is relevant to the KB. This p-value measure is corrected for multiplicity testing and the FDR is used as a score for relevance. Concepts with an FDR > 0.05 are cut off from the annotation, and are not used when enriching concept pairs/associations.

#### Pair Enrichment

**Figure 5:**
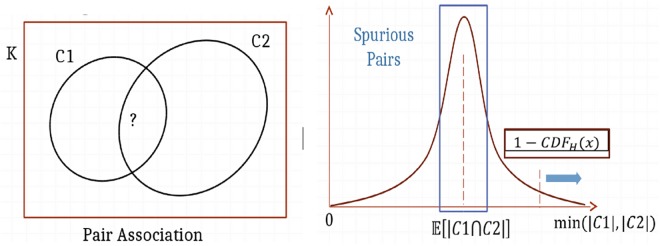
Pair Enrichment in DES.

Pair enrichment is performed in exactly the same manner (see Fig. 5), by considering hits against one of the concepts as taking a biased sample, then performing statistical enrichment of the other concept against this sample, as explained above. The statistical significance is again established by cutting off any pairs having an FDR > 0.05.

So, in conclusion: statistical significance in both cases is determined using a threshold on the FDR value.

Measures used for evaluating DES mutation annotation compared to EMU:

1 $$Precision=\frac{TP}{TP+FP}\,$$

2 $$Recall=\frac{TP}{TP+FN}$$

3 $$F\_measure=2\,\times \frac{precision\times recall}{precision+recall}$$

Precision is the proportion of valid instances (true positives) among all detected instances (both true and false positives). It was used for our comparison with EMU as a measure of the quality of mutations extracted by DES-mutation from a randomly selected sample of 1000 abstract-only documents. Recall is the proportion of valid instances extracted (true positives) over all valid instances (both true positives and false negatives). It was used for our comparison with EMU as a measure of the extent of coverage of DES-Mutation of the mutations mentioned in the randomly selected sample of 1000 abstract-only documents. F-measure combines both precision and recall and is computed as their harmonic mean.

## Electronic supplementary material

Supplementary Table S1

## Data Availability

The DES-Mutation portal is free for academic and nonprofit users and can be accessed at http://cbrc.kaust.edu.sa/des-mutation/.
